# Supplementary material for: Hydrogen Selective SiCH Inorganic–Organic Hybrid/γ-Al2O3 Composite Membranes
Source: Membranes (Basel). 2020 Sep 25;10(10):258. doi: 10.3390/membranes10100258 (PMC7600925; doi:10.3390/membranes10100258)
Supplement: Supplementary file 1 [file membranes-10-00258-s001.pdf]

membranes

Electric Supplementary Information

Hydrogen Selective SiCH Inorganic-Organic Hybrid/γ-Al_2O_3 Composite Membranes

Miwako Kubo[1], Ryota Mano[1], Misako Kojima[1], Kenichi Naniwa[1], Yusuke Daiko[1], Sawao Honda[1], Emanuel Ionescu[2], Samuel Bernard[3], Ralf Riedel[2] and Yuji Iwamoto[1*]

[1] Department of Life Science and Applied Chemistry, Graduate School of Engineering, Nagoya Institute of Technology, Gokiso-cho, Showa-ku, Nagoya 466-8555, Japan; m.kubo.579@stn.nitech.ac.jp (M.K.); r.mano.012@nitech.jp (R.M.); m.kojima.811@stn.nitech.ac.jp (M.K.); k.naniwa.270@stn.nitech.ac.jp (K.N.); daiko.yusuke@nitech.ac.jp (Y.D.); honda@nitech.ac.jp (S.H.); iwamoto.yuji@nitech.ac.jp (Y.I.)

[2] Institut für Materialwissenschaft, Technische Universität Darmstadt, Otto-Berndt-Str. 3, 64287 Darmstadt, Germany; ionescu@materials.tu-darmstadt.de (E.I.), ralf.riedel@tu-darmstadt.de (R.R.)

[3] University of Limoges, CNRS, IRCER, UMR 7315, F-87000, Limoges, France; samuel.bernard@unilim.fr (S.B.)

* Correspondence: iwamoto.yuji@nitech.ac.jp; Tel.: +81-52-735-5276

Heat treatment temperatures selected for SiCH organic-inorganic hybrid synthesis

In this study, allyl-hydro-polycarbosilane (AHPCS) was converted to SiCH organic-inorganic hybrid as a component of hydrogen separation membrane. The temperatures for thermal conversion of AHPCS to SiCH hybrid in this study was selected as 300, 400 and 500 °C based on the results obtained by the simultaneous thermogravimetric (TG)-mass spectrometry (MS) analyses shown in Figures S3 and S4, Fourier transform (FT)-IR and Raman spectroscopic analyses for the heat-treated AHPCSs shown in Figures S5 and S6, respectively.

Characterizations

The molecular weight distribution curve of as-received AHPCS was measured at 40 °C by using Gel Permeation Chromatography (GPC, Model ShodexGPC-104 equipped with two tandem columns (Model Shodex LF-404) and a refractive index detector (Model Shodex RI-74S), Showa Denko K.K., Tokyo, Japan). The columns were calibrated against polystyrene standards. Tetrahydrofuran (THF) was used as the eluent and a flow rate was adjusted to 1.0 mL min^{-1}.

The thermal decomposition and cross-linking behaviors of as-received AHPCS up to 1000 °C was studied by thermogravimetry combined with mass spectrometry (TG-MS) analyses (Model STA7200, Hitachi High Technologies Ltd., Tokyo, Japan/Model JMS-Q1500 GC, JEOL, Tokyo, Japan). The measurements were performed under helium (He) atmosphere with a heating rate of 10 °C min^{-1}.

Fourier transform (FT)-IR spectrum was recorded on the as-received AHPCS and AHPCS-derived powder samples by the potassium bromide (KBr) disk method (Model FT/IR-4200IF, JASCO Corp., Tokyo, Japan).

The Raman spectrum was recorded on as-received AHPCS and heat-treated AHPCS (Renishaw, inVia Reflex, England).

Powder samples of the heat-treated AHPCS were prepared by heat treatment at 300, 400 and 500 ˚C under argon (Ar). Note that, FT-IR spectrum was also recorded on the powder sample of 700 °C-heat treated AHPCS.

Results and Discussion

Chemical structure and molecular distribution of the AHPCS are shown in Figures S1 and S2, respectively. The thermal behavior of AHPCS has been already studied by several research groups [1-4], and the results obtained in this study were well consistent with those previously reported: As shown in Figure S2, as-received AHPCS had a considerable amount of low molecular weight fraction below 1000. TG-MS analyses revealed the thermal decomposition of the low molecular weight fraction proceeded during the first weight loss at 100 to 300 °C and second one from 350 to 500 °C by detecting gaseous species assigned to the fragments of carbosilane species (Figures S3 and S4). On the other hand, thermal cross-linking was observed up to 300 °C for formation of ≡Si-CH_2-CH_2-CH_2-Si≡ and/or ≡Si-CH(CH_3)-CH_2-Si≡ via hydrosilylation between ≡Si-H and ≡Si-CH_2-CH=CH_2 groups in AHPCS, which was identified by the disappearance of the FT-IR absorption band at 1629 cm^{-1} attributed to C=C bond of allyl group [2,3] associated with the decrease in the relative FT-IR band intensities assigned to ν(Si-H) at 2123 cm^{-1} and δ(Si-H) at 947 cm^{-1} [2,3] (Figure S5).

At 400 to 700 °C, formation of ≡Si-Si≡ by the reaction between Si-H and Si-CH_3 groups was suggested by detecting the m/z ratio at 15 assigned to methane (CH_4) (Figure S4(b)). Because the thermal decomposition and cross-linking contentiously proceeded at 300 to 500 °C, the quantity of organic groups and microporosity of the SiCH hybrid differed depending on the specific heat treatment temperature in this temperature range. On the other hand, the FT-IR spectrum of the 700 °C-heat treated AHPCS revealed that polymer/inorganic silicon carbide conversion almost completed (Figure S5). It should be noted that the samples heat-treated at 300 to 500 °C were SiCH hybrid without graphite-like carbon, since the Raman spectra of these samples exhibited several peaks due to the organic-groups without those attributed to graphite-like carbon typically detected at 1347.5 and 1596.5 cm^{-1} assigned as D-band (for disordered graphite) and G-band (for the sp2 graphite network), respectively [5,6] (Figure S6). Based on these results, heat treatment of as-received AHPCS in this study was performed at 300, 400 and 500 °C for the synthesis of powder and membrane samples.

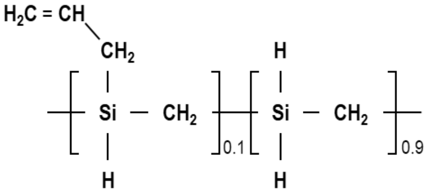

Figure S1. Structure of commercially available allyl-hydrido-polycarbosilane (AHPCS).

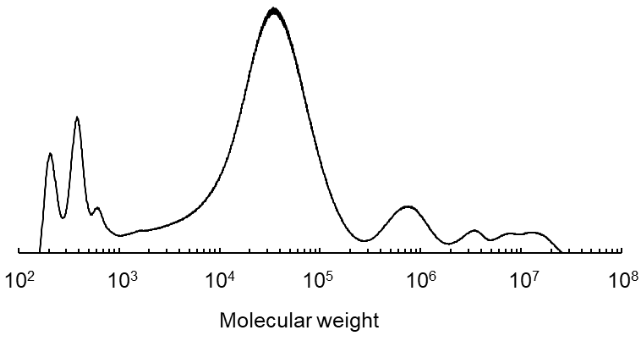

Figure S2. Molecular weight distribution of as-received AHPCS.

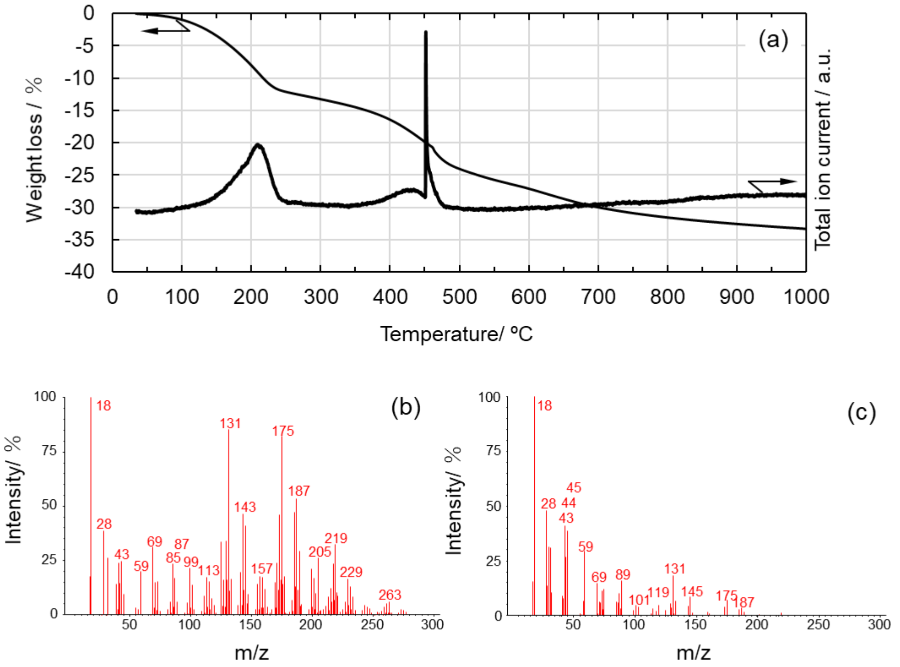

Figure S3. Thermal behavior of as-received AHPCS. (a) TG curve and total ion current chromatogram (TICC) under flowing He, and typical mass spectra recorded during (b) the first weight loss from100 to 250 °C and (c) the second weight loss from 350 to 500 °C.

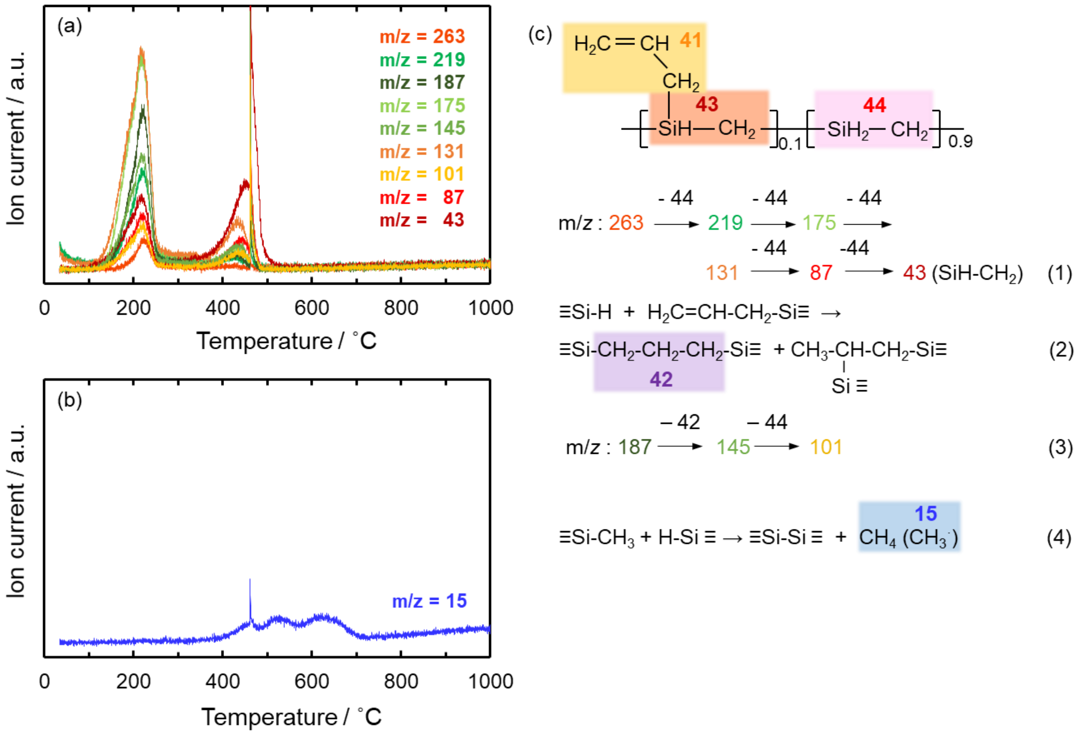

Figure S4. Continuous in-situ monitoring of gaseous species by mass spectrometry: (a) Fragments derived from low molecular weight fraction of as-received AHPCS during the first weight loss from 100 to 300 °C and the second one from 350 to 500 °C and (b) methane (CH_4) at 400 to 700 °C. (c) Fragments suggested for gaseous spices derived from low molecular weight fraction of as-received AHPCS (Eq. 1), m/z= 42 ($(CH_2)_3$) from AHPCS after

cross-linking via hydrosilylation between ≡Si-H and CH_2=CH-CH_2-Si≡ (Eqs. 2 and 3) and m/z= 15 (CH_4) due to the thermal crosslinking between ≡Si-H and ≡Si-CH_3 groups to afford ≡Si-Si≡ above 350 °C (Eq. 4) [4].

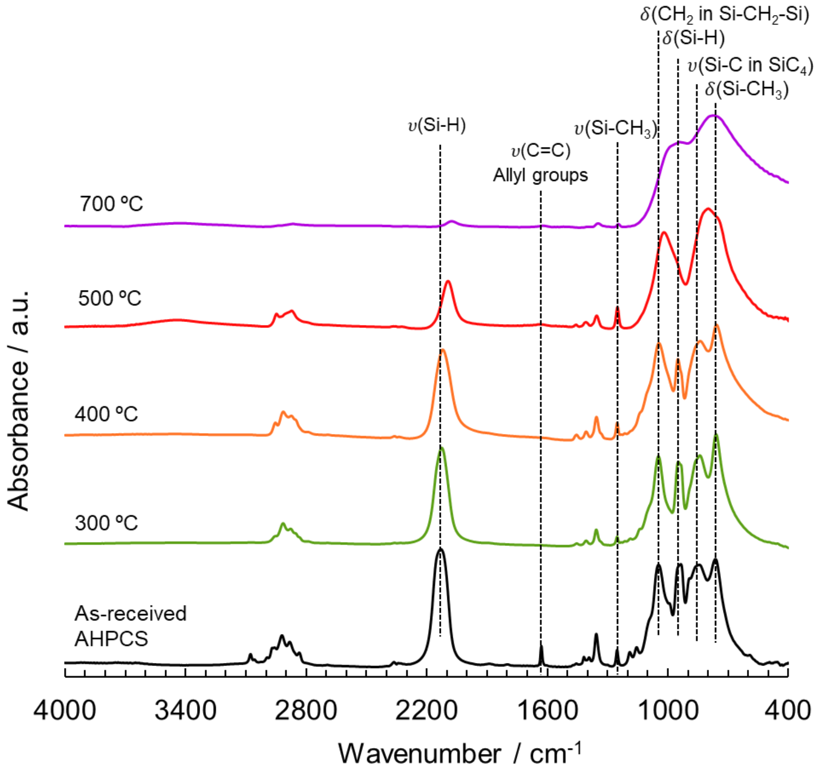

Figure S5. FT-IR spectra for as-received AHPCS and those after heat treatment at 300 to 700 °C in Ar.

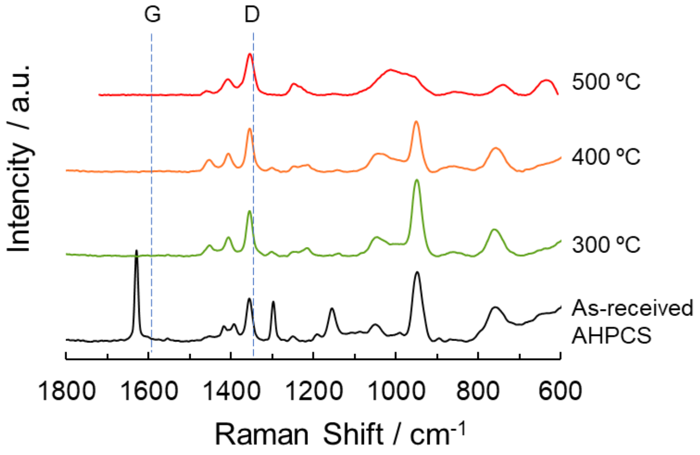

Figure S6. Raman spectra for as-received AHPCS and those after heat treatment at 300 to 500 °C in Ar. Spectra indicated the heat-treated samples were free from graphite-like carbon typically detected

at 1347.5 and 1596.5 cm^{-1} attributed to the D-band (for disordered graphite) and G-band (for the sp2 graphite network), respectively [5,6].

References

1. Sreeja, R.; Swaminathan, B.; Painuly, A.; Sebastian, T. V.; Packirisamy, S. Allylhydridopolycarbosilane (AHPCS) as matrix resin for C/SiC ceramic matrix composites. *Mater. Sci. Eng. B Solid-State Mater. Adv. Technol.* **2010**, *168*, 204–207, doi:10.1016/j.mseb.2009.12.033.
2. Kaur, S.; Riedel, R.; Ionescu, E. Pressureless fabrication of dense monolithic SiC ceramics from a polycarbosilane. *J. Eur. Ceram. Soc.* **2014**, *34*, 3571–3578, doi:10.1016/j.jeurceramsoc.2014.05.002.
3. Wen, Q.; Xu, Y.; Xu, B.; Fasel, C.; Guillon, O.; Buntkowsky, G.; Yu, Z.; Riedel, R.; Ionescu, E. Single-source-precursor synthesis of dense SiC/HfC_xN_{1-x}-based ultrahigh-temperature ceramic nanocomposites. *Nanoscale* **2014**, *6*, 13678–13689, doi:10.1039/c4nr03376k.
4. Wang, Q.; Yokoji, M.; Nagasawa, H.; Yu, L.; Kanezashi, M.; Tsuru, T. Microstructure evolution and enhanced permeation of SiC membranes derived from allylhydridopolycarbosilane. *J. Memb. Sci.* **2020**, 118392, doi:10.1016/j.memsci.2020.118392.
5. Ferrari, A.C.; Robertson, J. Interpretation of Raman spectra of disordered and amorphous carbon. *Phys. Rev. B* **2000**, *61*, 14095–14107, doi:10.1103/PhysRevB.61.14095.
6. Pimenta, M.A.; Dresselhaus, G.; Dresselhaus, M.S.; Cançado, L.G.; Jorio, A.; Saito, R. Studying disorder in graphite-based systems by Raman spectroscopy. *Phys. Chem. Chem. Phys.* **2007**, *9*, 1276–1291, doi:10.1039/b613962k.

© 2020 by the authors. Submitted for possible open access publication under the terms and conditions of the Creative Commons Attribution (CC BY) license (http://creativecommons.org/licenses/by/4.0/).
